# Comparison of the Degradation Effect of Methylene Blue for ZnO Nanorods Synthesized on Silicon and Indium Tin Oxide Substrates

**DOI:** 10.3390/ma16124275

**Published:** 2023-06-09

**Authors:** Guoxiang Peng, Ni-Ni Chou, Yu-Shan Lin, Cheng-Fu Yang, Teen-Hang Meen

**Affiliations:** 1 School of Ocean Information Engineering, Jimei University, Xiamen 361021, China; gxpeng@jmu.edu.cn; 2 Department of Chemical and Materials Engineering, National University of Kaohsiung, Kaohsiung 811, Taiwan; a1091402@mail.nuk.edu.tw (N.-N.C.); 4134isha@gmail.com (Y.-S.L.); 3 Department of Aeronautical Engineering, Chaoyang University of Technology, Taichung 413, Taiwan; 4 Department of Electronic Engineering, National Formosa University, Yunlin 632, Taiwan

**Keywords:** ZnO nanorods, different substrates, indium tin oxide, photocatalysis, degradation, methylene blue

## Abstract

In the context of ZnO nanorods (NRs) grown on Si and indium tin oxide (ITO) substrates, this study aimed to compare their degradation effect on methylene blue (MB) at different concentrations. The synthesis process was carried out at a temperature of 100 °C for 3 h. After the synthesis of ZnO NRs, their crystallization was analyzed using X-ray diffraction (XRD) patterns. The XRD patterns and top-view SEM observations demonstrate variations in synthesized ZnO NRs when different substrates were used. Furthermore, cross-sectional observations reveal that ZnO NRs synthesized on an ITO substrate exhibited a slower growth rate compared to those synthesized on a Si substrate. The as-grown ZnO NRs synthesized on the Si and ITO substrates exhibited average diameters of 110 ± 40 nm and 120 ± 32 nm and average lengths of 1210 ± 55 nm and 960 ± 58 nm, respectively. The reasons behind this discrepancy are investigated and discussed. Finally, synthesized ZnO NRs on both substrates were utilized to assess their degradation effect on methylene blue (MB). Photoluminescence spectra and X-ray photoelectron spectroscopy were employed to analyze the quantities of various defects of synthesized ZnO NRs. The effect of MB degradation after 325 nm UV irradiation for different durations can be evaluated using the Beer–Lambert law, specifically by analyzing the 665 nm peak in the transmittance spectrum of MB solutions with different concentrations. Our findings reveal that ZnO NRs synthesized on an ITO substrate exhibited a higher degradation effect on MB, with a rate of 59.5%, compared to NRs synthesized on a Si substrate, which had a rate of 73.7%. The reasons behind this outcome, elucidating the factors contributing to the enhanced degradation effect are discussed and proposed.

## 1. Introduction

In addition to being used in the textile industry, dyes have various applications in everyday life, such as in rubber, plastic, printing, and even food production [1]. However, the treatment of dyeing and finishing wastewater is a complex task, leading to environmental concerns. Consequently, large volumes of dye wastewater are produced, posing an increasing threat to the environment as a significant source of pollution. Methylene blue (MB) is a phenothiazine salt with the chemical formula C_16_H_18_N_3_ClS. It appears as a dark green bronze lustrous crystal or powder and can dissolve in water and ethanol, but not in ethers. MB finds its application in chemical indicators, dyes, biological stains, and medicines. It has relatively good stability in air, but its aqueous solution is alkaline and toxic. Due to its stability and resistance to fading, the MB is popularly used in garment textile dyeing. On 27 October 2017, the International Agency for Research on Cancer of the World Health Organization compiled a list of carcinogens, which included MB as one of the three types. Recently, there has been intensive study of techniques for removing dyes due to increasingly strict international environmental standards. In recent years, there has been a growing awareness of the importance of environmental protection. As a result, scientists have devoted significant resources to researching solutions to address the issue of pollution in the environment. Therefore, in order to solve the problem of dye wastewater, many methods have been investigated to generally treat the dye wastewater, including advanced oxidation technologies, chemical decomposition, biological processes, photocatalysis, etc.

For example, Wei et al. (2019) investigated the utilization of a nanoceria/H_2_O_2_ system in alkaline conditions to overcome the limited adsorption of MB on the nanoceria surface caused by electrostatic attraction [2]. This system demonstrated efficient adsorption and degradation of MB. In another study, Vanaja et al. successfully synthesized silver nanoparticles using Morinda tinctoria leaves as a source, at various pH levels. They then employed the synthesized silver nanoparticles to effectively degrade MB under sunlight irradiation [3]. Minamoto et al. discovered that the discoloration of MB caused by air microbubbles provides evidence for the generation of hydroxyl radicals through microbubble collapse in water. Furthermore, they observed that the decomposition rate of MB escalates with the concentration of acids (such as H_2_SO_4_, HNO_3_, and HCl) and H_2_O_2_, up to approximately 2 mM [4]. Thus, the most effective method for decomposing organic pollutants in water is through photocatalytic treatment using a photocatalyst [5]. Fujishima (1972) discovered the photocatalytic properties of titanium dioxide. When exposed to ultraviolet light, titanium dioxide can break down water molecules and produce hydrogen gas, thereby revealing its photochemical properties [6]. Other materials, such as oxides including TiO_2_, ZnO, SnO_2_, and ZrO_2_, as well as sulfides such as CdS and ZnS, are also commonly used as photocatalysts.

By harnessing photocatalytic reactions, the energy from photons can be converted into chemical energy. This energy is utilized to oxidize and decompose organic pollutants present in the wastewater, transforming them into harmless compounds such as carbon dioxide, water, and inorganic salts. This process proves to be exceptionally efficient at eliminating color from dye wastewater and presents numerous advantages, including potent oxidative capability, energy efficiency, rapid reaction rates, simplicity in operation, and cost-effectiveness. As a result, it has garnered significant attention and undergone extensive research in many countries around the world [7]. For example, the efficient catalytic ability of 3-D structures of copper sulfide (CuS) is attributed to its high surface-area-to-volume ratio, which facilitates better contact between reactants and CuS. Additionally, CuS is an ideal candidate for MB degradation due to its stable nature under ambient condition, and it is non-toxic and cost-effective [8]. Chen et al. utilized the anodic oxidation technique to cultivate arrays of TiO_2_ nanotubes (TNAs) on Ti metal substrate. The TNAs were then subjected to annealing within the temperature range of 350~600 °C [9]. The annealed TNAs were employed as photocatalysts for the degradation of MB, whereby the TNAs annealed at 500 °C, which contained only the anatase phase, exhibited the most effective MB degradation results. 

In the past, ZnO-based nanomaterials were found to possess photocatalytic properties capable of degrading MB in water, and various ZnO-based materials have been utilized as photocatalysts for the degradation of MB. Numerous ZnO-based nanoparticles, nanocomposites, and compounds formed by mixing with different materials have also been utilized as photocatalysts for the degradation of MB [10,11,12]. Similarly, a nanostructured ZnO film deposited on a Si substrate using reactive magnetron sputtering was utilized as a photocatalyst for the degradation of MB [13]. ZnO-based nanorods (NRs) are also an important nanomaterial that can be used to degrade MB [14]. For example, Zhao et al. synthesized ZnO NRs on a patterned cavity sapphire substrate to form ZnO nanoflower arrays, and they used the growth ZnO nanoflower arrays to degrade MB [15]. In the past, the most commonly used substrate for depositing a ZnO seed layer was silicon. Due to ZnO’s capability to degrade MB, numerous studies aim to enhance its degradation effectiveness. Most of the recent studies of ZnO NRs have primarily concentrated on investigating the impacts of various dopant impurities on their physical properties and their efficiency of MB degradation [16]. For instance, Khalid et al. (2019) conducted a study where they co-doped ZnO NRs with Al and Fe. They observed that the Al and Fe co-doped ZnO NRs exhibited a seventeen-fold higher degradation rate constant for MB dye compared to pure ZnO [17]. In another study, Akhtar et al. (2020) investigated the co-doping of ZnO NRs with Gd and Nd. They discovered that the optimized 1.5% Nd/ZnO nanocomposite exhibited significantly enhanced photocatalytic performance for the degradation of methylene blue in comparison to pure ZnO and other nanocomposites [18]. However, to date, only a limited number of studies have utilized the hydrothermal method to synthesize ZnO NRs on indium tin oxide-glass (abbreviated as ITO) substrate; however, there has never been a study on the degradation of MB using ZnO NRs grown on an ITO substrate.

E-beam technology can offer several advantages for the deposition of a ZnO seed layer, including the allowance for fast and efficient processing, being easily customized and adaptable to specific applications, and high reproducibility, stability, and accuracy for the thickness control. Thus, E-beam evaporation was employed to deposit a ZnO seed layer on two different types of substrates. Next, ZnO NRs were synthesized using the two types of substrates. ZnO NRs possess photocatalytic properties, leading to their widespread utilization by researchers in the field of dye degradation in recent years. Previously, we have successfully grown ZnO NRs on various substrates, including an ITO substrate. Our experimental data indicate that ZnO NRs deposited on an ITO substrate exhibit distinct optical properties when compared to those deposited on a Si substrate. Notably, ZnO NRs grown on an ITO substrate exhibit significantly stronger and photoluminescence emission (PL) intensities. Thus, the first significant novelty of this study is the discovery that ZnO NRs synthesized on distinct substrates exhibit varied surface morphologies, crystalline phases, and PL properties. The second significant novelty pertains to the photo-catalytic activities of ZnO NRs synthesized on two different substrates, which were evaluated through experiments on MB degradation under UV-light irradiation. 

The experimental results demonstrate that ZnO NRs synthesized on an ITO substrate displayed a greater degradation effect compared to those synthesized on a Si substrate. In any case, our findings indicate that the degradation rate of MB dye by ZnO NRs grown on an ITO substrate was higher compared to ZnO NRs grown on a Si substrate. Additionally, it was observed that the efficiency of the MB decreased by more than 50% after being exposed to ZnO NRs synthesized on an ITO substrate for 100 min. SEM observations revealed that ZnO nanorods (NRs) synthesized on a Si substrate exhibited a greater growth rate. Additionally, BET measurements indicated that ZnO NRs synthesized on a Si substrate possessed a larger specific surface area compared to those synthesized on an ITO substrate. Based on these findings, it can be inferred that the enhanced degradation effect of ZnO NRs on MB is not solely attributed to the specific surface area. However, the specific factors contributing to this difference have not been extensively investigated in previous research. Therefore, this study aims to propose a potential explanation for the higher degradation effect observed in ZnO NRs synthesized on an ITO substrate. As a result, the third significant contribution of this study is the analysis of both PL spectra and X-ray photoelectron spectroscopy (XPS) spectra, which were utilized to identify the factors responsible for the superior degradation effect on MB achieved through the synthesis of ZnO NRs on an ITO substrate compared to a Si substrate.

## 2. Materials and Methods

Firstly, the Si and ITO substrates were cut to a 20 × 20 mm^2^ size. The substrates underwent a series of fixed cleaning processes to eliminate any organic matter and impurities on the surface. This involved using an ultrasonic cleaner, deionized water, acetone, and isopropanol. Next, anhydrous alcohol was used to rinse any residual moisture on the substrates, followed by drying the surfaces of the silicon substrate and ITO glass substrate with nitrogen to complete the cleaning processes. Once the cleaning processes were completed, a ZnO seed layer was deposited on both the Si and ITO glass substrates using the electron beam evaporation method (FSE corporation, Hsinchu, Taiwan). A ZnO ceramic body was utilized as the target material for depositing a ZnO seed layer. Initially, the chamber pressure was evacuated to a value of 7.8 × 10^−6^ torr. Once the deposition process commenced, the chamber pressure was maintained at a constant level of 1 × 10^−5^ torr. The deposition rate was set at 0.1 nm/s, while the desired thickness for the seed layer was 30 nm. It is noteworthy that the electron beam evaporation method was employed for depositing ZnO seed layer due to its high reproducibility, stability, and accuracy. To prepare the solution containing 0.2 M Zn^2+^ ions, ethylene glycol monomethyl ether (CH_3_OCH_2_CH_2_OH), monoethanolamine (C_2_H_7_NO), and Zinc nitrate hexahydrate (Zn(NO_3_)_2_·6(H_2_O)) were utilized. The solution was then used to synthesize ZnO NRs on both silicon (Si) substrate and ITO glass substrate, which were held at a temperature of 100 °C for 3 h. The growth time of three hours is necessary to achieve the desired length, while a temperature of 100 °C confirms the successful growth of ZnO NRs. If the temperature is too low, ZnO NRs will not be able to grow. The silicon substrate primarily utilized a p-type substrate with approximately 10 nm SiO_2_ on its surface. On the other hand, for ITO, a substrate with a low resistivity of 10^−4^ Ω.cm was employed.

After the growth of ZnO NRs on various substrates, their crystalline phases were analyzed using an X-ray diffractometer, while their surface and cross-sectional morphologies were observed using a field-effect scanning microscope system (SEM). The crystallization of ZnO NRs was also observed using a high resolution transmission electron microscopy (HRTEM). The BET (Brunauer–Emmett–Teller) analysis is recognized an effective technique to find the specific surface area of nanomaterials. Therefore, the BET analysis was used to measure the specific surface areas of ZnO NRs synthesized on different substrates. To analyze their photoluminescence (PL) spectra at room temperature across the 350–650 nm wavelength range, a Horiba Jobin iHR550 fluorescence spectrophotometer was utilized, which employed a He-Cd laser with a single wavelength of 325 nm as the excitation light source. The experiments to synthesize ZnO NRs on both Si and ITO substrates were conducted to investigate their effectiveness in degrading MB. To achieve this, a 5 ppm MB solution was prepared and 20 × 20 mm^2^ ZnO NRs with different substrates were immersed in 5 mL of the solution. 

The MB solution and ZnO NRs were subsequently exposed to 365 nm ultraviolet light with a power density of 720–760 μw/cm^2^ (UVGL-25, Science Company, Lakewood, CO, USA) for different durations, ranging from 0 to 100 min. To evaluate the degradation efficiency of MB in the liquid, the Beer–Lambert law was used to analyze the average value of five data points to measure the concentration variations of the MB solution. Additionally, a Hitachi U-3300 UV-Vis spectrophotometer was used to measure the optical transmittance spectra of the MB solutions across the wavelength range of 200–700 nm.

## 3. Results and Discussion

**Figure 1:**
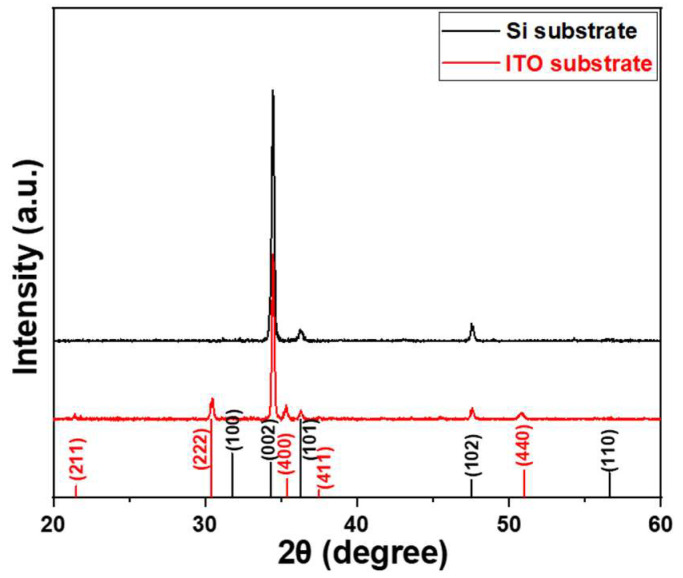
XRD patterns of NRs synthesized on Si and ITO substrates. Black indexes are used for ZnO diffraction peaks and red indexes are used for ITO diffraction peaks. ZnO NRs were synthesized at a temperature of 100 °C for 3 h.

The diffraction patterns of ZnO NRs synthesized on Si and ITO glass substrates were analyzed, and the results are presented in Figure 1. The JCPDS 36-1451 and 71-2194 cards (The Joint Committee on Powder Diffraction Standards) for ZnO and ITO are also shown in Figure 1 to compare the diffraction peaks of ZnO NRs on different substrates. For ZnO NRs synthesized on the two different substrates, the (002) plane exhibited the highest intensity peak at 34.4° for both substrates, and it was more prominent than the other diffraction peaks. Figure 1 demonstrates that the diffraction intensity of the (002) plane is greater for ZnO NRs synthesized on a Si substrate than for those synthesized on an ITO substrate. ZnO NRs synthesized on both Si and ITO substrates exhibited a preferred orientation along the *c*-axis, as evidenced by the high diffraction intensities of the (002) plane. This preference is due to the perpendicular growth of ZnO NRs to the Si and ITO substrates, which allows them to grow vertically. However, some weaker peaks differ from previous studies where ZnO NRs had apparent peaks at (100), (101), (102), and (110) planes. In our study, only the (101) and (102) planes were observed for ZnO NRs synthesized on a Si substrate, while ZnO NRs synthesized on an ITO substrate showed the (100), (101), and (102) planes. The differences in diffraction intensity and peak patterns suggest that the choice of substrate is a crucial factor that influences the crystal properties of synthesized ZnO NRs. Additionally, the diffraction peaks for ITO (211), (222), (400), (411), and (440) planes are also shown Figure 1. 

The observed crystallographic planes (002), (101), and (102) for ZnO NRs synthesized on a Si substrate, as well as the additional planes (100), (002), (101), and (102) for ZnO NRs synthesized on an ITO substrate, hold significance in understanding the structural properties and behavior of synthesized ZnO NRs. Crystallographic planes of ZnO NRs refer to the specific arrangement of atoms within a crystal lattice. By analyzing the observed crystallographic planes, researchers can gain insights into the orientation, quality, and growth characteristics of ZnO NRs. The plane (002) is associated with the *c*-axis orientation, which indicates the growth direction of ZnO NRs. The (101) and (102) planes provide information about the crystallinity and the specific facet orientations of ZnO NRs. These facets play a crucial role in determining the surface reactivity and catalytic properties of ZnO NRs. In the case of ZnO NRs synthesized on the Si substrate, the presence of (002), (101), and (102) planes indicates the growth of vertically aligned ZnO NRs, which are well-oriented along the *c*-axis. This alignment is desirable for various applications such as optoelectronics, sensors, and solar cells. On the other hand, for ZnO NRs synthesized on an ITO substrate, the additional (100) plane suggests a different growth behavior compared to ZnO NRs synthesized on a Si substrate. The presence of (002), (101), and (102) planes alongside (100) indicates the formation of ZnO NRs on ITO substrate with a more complex crystal structure, which may cause by the formation of more defects in ZnO NRs synthesized on an ITO substrate.

**Figure 2:**
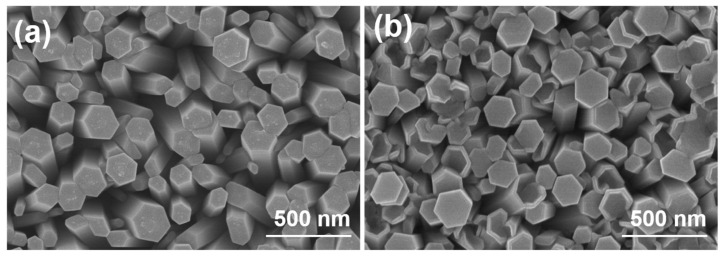
ZnO NRs synthesized on (**a**) Si and (**b**) ITO substrates. ZnO NRs were synthesized at a temperature of 100 °C for 3 h.

Figure 2a,b shows a top view of ZnO NRs that are aligned on a Si substrate and an ITO substrate. ZnO NRs synthesized on two different substrates exhibit the consistently hexagonal nanorods along their lengths, indicating a hexagonal crystal structure and preferential growth along the *c*-axis direction. The as-grown ZnO NRs synthesized on Si and ITO substrates had the average diameters of 110 ± 40 and 120 ± 32 nm and had the average lengths (not shown here) of 1210 ± 55 and 960 ± 58 nm, respectively. It is challenging to determine the exact reason behind the lower growth rate of ZnO NRs synthesized on an ITO substrate. However, a possible explanation will be proposed for this outcome later. Due to the high density of vertically grown ZnO nanorods (NRs) across the entire substrate surface, both substrates can be effectively used for large-scale production of aligned ZnO NRs. Furthermore, these NRs show great potential as photocatalysts for MB degradation in future applications. Notably, Figure 2 demonstrates the synthesis of ZnO NRs on a Si substrate, where the tops retain a hexagonal shape. However, when synthesized on an ITO substrate, although some NR tops present the perfectly hexagonal shape, a significant portion of them exhibits defects in their hexagonal surface structure. These results demonstrate a higher occurrence of defects in ZnO NRs synthesized on an ITO substrate compared to those synthesized on a Si substrate. 

The findings indicate that the crystallization properties of ZnO NRs can vary depending on the substrate utilized, as depicted in Figure 1. Furthermore, these results suggest that the efficiency of MB degradation by ZnO NRs could vary depending on the substrate on which they are synthesized. The specific surface areas of ZnO NRs grown on different substrates were determined using the BET method. The measurements yielded values of 16.8 m^2^/g and 13.3 m^2^/g when Si and ITO were used as the substrates, respectively. Based on our hypothesis, the increased specific surface area observed in ZnO NRs synthesized on a Si substrate is attributed to their longer length. The thermal effect induces a dehydration reaction, resulting in the formation of a solid-phase product that grows along the crystal structure of ZnO. The growth process of ZnO nanomaterials primarily involves two main steps: a hydrolysis reaction and a dehydration reaction. The chemical reaction formula for this process can be expressed as follows [19,20]:

C_6_H_12_N_4_ + 6H_2_O → 4NH_3_ + 6HCHO (1)

4NH_3_ + H_2_O ↔ OH^−^ + NH_4_^+^ (2)

Zn^2+^ + OH^−^ ↔ Zn(OH)_2_ ↔ H_2_O + ZnO(s) (3)

The equilibrium reaction formula indicates that the direction of the reaction can be influenced by the concentration of reactants, growth temperature, and growth time [19,20]. In general, the concentration of reactants determines the density of the one-dimensional ZnO nanorod structure. Meanwhile, the growth time and heating temperature can regulate the morphology and aspect ratio of ZnO nanorods. However, the specific reasons for the higher defect density and slower growth of ZnO nanorods on indium tin oxide (ITO) are not well understood. For the ITO film, after doping In_2_O_3_ with Sn, the Sn atoms can substitute the In atoms within the In_2_O_3_ crystal structure, resulting in the formation of SnO_2_. Due to the trivalent nature of the In atoms in In_2_O_3_, an electron is donated to the conduction band upon the formation of SnO_2_. This process leads to the generation of oxygen vacancies, resulting in the creation of oxygen holes. In an oxygen-deficient state, this leads an ITO substrate to have a high carrier (electron) concentration ranging from 10^20^ to 10^21^ cm^−3^. When a ZnO seed layer is deposited on an ITO substrate, the conductivity of the resulting ZnO film may not be optimal. However, the electrons present in the ITO film will still be transported through the seed layer to the location where the ZnO NRs are intended to grow. Consequently, the OH^−^ ions will experience reduction, thereby impeding the growth of ZnO NRs. As a result, the growth of ZnO NRs on ITO is characterized by increased defect formation and a slower growth rate.

**Figure 3:**
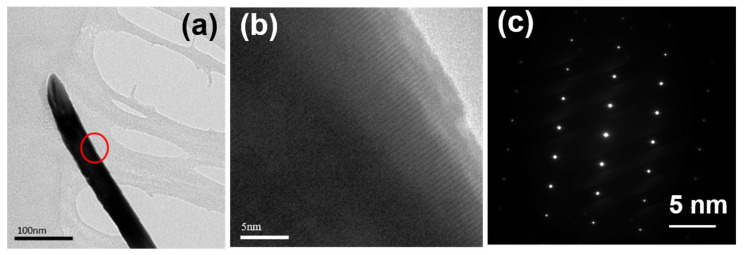
TEM observation of ZnO NRs synthesized on a Si substrate and at a temperature of 100 °C for 3 h. (**a**) A ZnO NR for TEM observation (red circle: observation), (**b**) HRTEM image and (**c**) electron diffraction pattern for a ZnO NR.

High-resolution (HR) electron microscopy, which was operated at an acceleration voltage of 200 kV and captured using a field emission gun, was utilized to conduct measurements on the lattice fringe widths. The TEM observation result of a ZnO NR synthesized on a Si substrate are shown in Figure 3a. Based on the bright field image in Figure 3a, it is evident that the entire ZnO nanorod turned black after diffraction. In Figure 3a, the red circle is employed for processing the HRTEM observation, leading to the successful observation of the lattice fringe of the ZnO NR, as depicted in Figure 3b. Figure 3c showcases the processed fast Fourier transform (FFT) of the corresponding image diffraction, exhibiting clearly distinguishable circular light spots. These light spots indicate that the crystal planes of the entire ZnO NR conform to the diffraction conditions presented in Figure 1, and the lattice spacing observed measures 0.259 nm.

**Figure 4:**
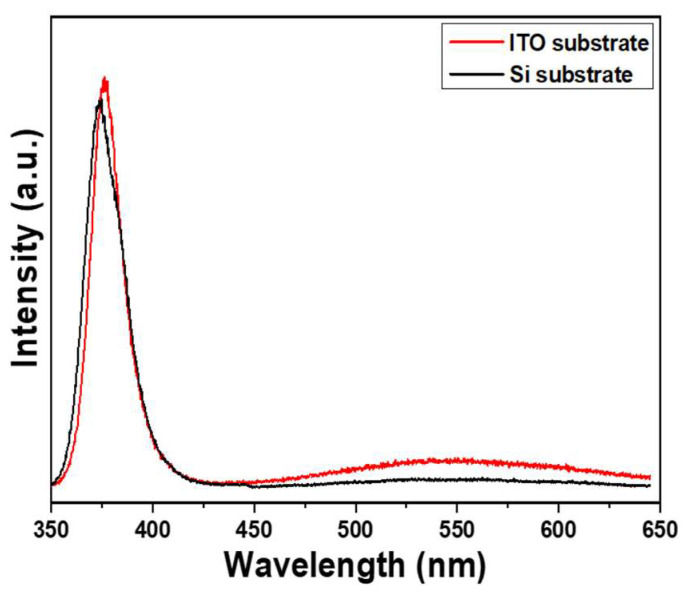
Effect of different substrates on the PL spectra of ZnO NRs on different substrates. ZnO NRs were synthesized at a temperature of 100 °C for 3 h.

Figure 4 shows the photoluminescence (PL) spectra of ZnO NRs on different substrates, excited using a 325 nm UV light. The spectra of ZnO NRs synthesized on both Si and ITO substrates exhibit two emission peaks: an ultraviolet (UV) emission peak centered at approximately 375 nm (referred to as IUV), and a green light emission (IG) ranging from approximately 450 to 550 nm. The IUV peak is a typical near-band-edge emission and arises from the recombination process of free excitons [21]. On the other hand, the broad band observed in the PL spectra corresponds to transitions between the local levels and near-band-edge, which are caused by intrinsic defects present in synthesized ZnO nanomaterials [22]. In previous researches, several luminescence mechanisms have been proposed for ZnO-based nanomaterials. For instance, Lin et al. identified various defects in ZnO-based nanomaterials, including zinc vacancies, interstitial zinc, antisite defects, interstitial oxygen, and oxygen vacancies [23]. These defects contribute to the emission of different types of light. Ultraviolet (UV) light emission occurs at approximately 390 nm (3.18 eV), while violet light emission, green light emission, and near-infrared emission occur at around 405 nm (3.06 eV), 414 nm (2.99 eV), 521 nm (2.38 eV), 544 nm (2.28 eV), and 765 nm (1.62 eV) respectively. Vanheusden et al. conducted a study on synthesized ZnO nanomaterials and found that the broad visible emission band observed in their experiments can be attributed to transitions between local levels and the near-band-edge. These transitions are caused by intrinsic defects present in the ZnO nanomaterials [24]. Similarly, Galdámez-Martinez et al. and Lin et al. observed electron transitions between deep levels and the valence band. These deep levels are associated with oxygen vacancies, impurities, Zn residues, and structural defects [25,26].

Figure 4 compares the emission spectra of ZnO NRs synthesized on two substrates. Figure 4 illustrates that the sharp peaks are located at 374.8 and 376.2 nm as Si and ITO were used as substrates. The results show that the ultraviolet (UV) emission peak intensities were similar for both substrates. However, ZnO NRs synthesized on an ITO substrate had a higher emission intensity of green light compared to those synthesized on a Si substrate. This suggests that ZnO NRs synthesized on a Si substrate have fewer defects, as indicated by their lower emission intensity in green light. In previous research conducted by El Filali et al., it was discovered that the decrease in intensity of the green photoluminescence (PL) band indicates the presence of zinc vacancies occupied by indium ions within the doping range of 0.5–2.5 at% [27]. They also found that this occupation of vacancies leads to a reduction in the concentration of inherent deep defects in ZnO, thereby enhancing the crystal quality of ZnO. Additionally, it results in a blue shift of the optical band gap, reaching a value of 3.31 eV. Therefore, the change in substrate material from Si to ITO leads to a red shift in the peak position of near-band-edge emission and an increase in visible light (green light) luminous intensity, both of which are attributed to the presence of additional defects in ZnO NRs synthesized on an ITO substrate. 

**Figure 5:**
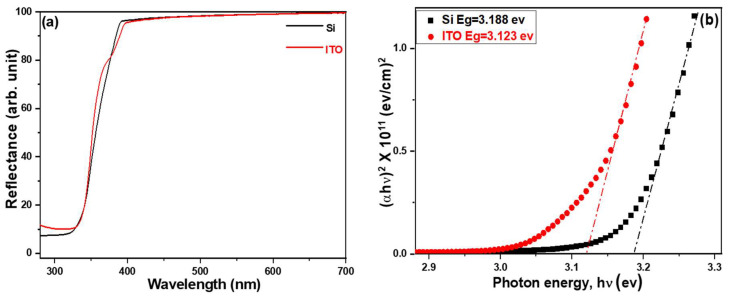
(**a**) UV–visible diffusion reflectance spectra and (**b**) Tauc plots of ZnO NRs on different substrates. ZnO NRs were synthesized at a temperature of 100 °C for 3 h.

Figure 5a illustrates the variations in the UV-visible diffusion reflectance spectra of ZnO nanorods (NRs) synthesized on different substrates. Meanwhile, Figure 5b shows the corresponding Tauc plots constructed using the diffusion reflectance spectra. The UV-visible diffusion reflectance spectra of ZnO NRs provide insights into the optical reflectance in the ultraviolet (UV) region, representing the transition of electrons from the valence band to the conduction band [22]. These spectra demonstrate that there were no significant differences in reflectance rates when different substrates were utilized for ZnO NRs synthesis. The calculated energy band gaps for ZnO NRs grown on Si and ITO substrates were 3.188 eV and 3.123 eV, respectively. When comparing the diffusion reflection spectra in Figure 5a, the reflection edge of ZnO NRs grown on a Si substrate exhibits a sharp and nearly linear change. In contrast, the reflectance edge of ZnO NRs grown on an ITO substrate does not follow a straight line but shows a slight turning point. This observation indirectly confirms the presence of minimal defects for ZnO NRs grown on a Si substrate, whereas some structural defects exist for ZnO NRs grown on an ITO substrate. 

Intrinsic defects in ZnO NRs include vacancies (missing Zn or O atoms) or interstitials (extra Zn or O atoms). These defects can create energy states within the band gap, commonly referred to as defect levels or trap levels. The positions of these defect levels within the band gap determine their influence on the energy gap of ZnO NRs. The energy gap of a pristine ZnO NR refers to the energy difference between its valence band and its conduction band. In the presence of defects, the defect levels introduced within the band gap can act as intermediate energy states. These states can trap charge carriers (electrons or holes) and affect the movement of these carriers, subsequently modifying the energy gap. Defects that introduce energy states closer to the conduction band are called shallow-level defects, while those closer to the valence band are referred to as deep-level defects. Shallow-level defects of ZnO NRs tend to increase the conductivity of ZnO NRs by facilitating the movement of charge carriers. Therefore, a decrease in the energy band gap of ZnO nanorods (NRs) can be considered as evidence of an increase in defects. Moreover, an increase in defects within ZnO NRs may potentially lead to a reduction in the growth rate.

**Figure 6:**
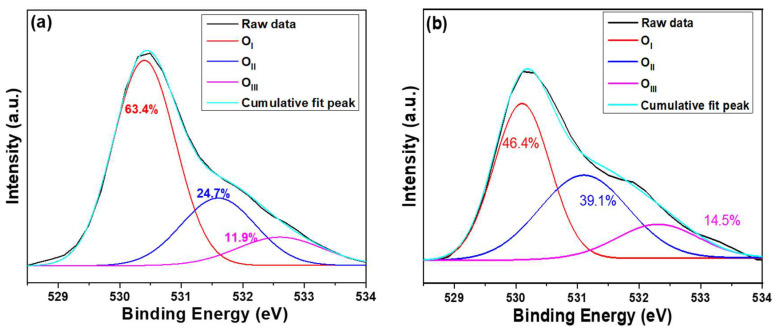
Drawings of O_1s_ peak of ZnO NRs on different substrates (**a**) on Si and (**b**) on ITO. ZnO NRs were synthesized at a temperature of 100 °C for 3 h.

To further investigate the impact of the used substrate on the properties of ZnO NRs, X-ray photoelectron spectroscopy (XPS) analysis was conducted to determine the oxygen chemical bonding state. The XPS data obtained can help to establish a correlation between the defects caused by different substrates and the optical properties of ZnO NRs. The O_1s_ peak of ZnO NRs synthesized on Si (ITO) substrate was analyzed, with the typical surface O_1s_ peak of ZnO NRs centered at 530.1 ± 0.2 (530.5 ± 0.2) eV. This peak was fitted using three Gaussian components corresponding to O_I_, O_II_, and O_III_ peaks, as Figure 6a,b shows. The O_I_, O_II_, and O_III_ peaks have peak positions at 530.1 ± 0.1 eV (530.4 ± 0.1), 531.1 ± 0.1 (531.6 ± 0.1) eV, and 532.3 ± 0.1 (532.6 ± 0.1) eV, respectively [28]. In ZnO NRs, the bond energies for the O_I_, O_II_, and O_III_ peaks arise from the Zn^2+^-O^2−^ bond, oxygen vacancies, and chemical adsorption of oxygen on the surfaces, respectively [28,29]. As ZnO NRs were synthesized on Si and ITO substrates, the areas of the O_I_ peak were 63.4% to 46.4%, the areas of the O_II_ peak were 24.1% and 39.1%, and the areas of the O_III_ peak were 11.9% and 14.5%, respectively. The area of the O_II_ peak for ZnO NRs synthesized on an ITO substrate was significantly larger than that on a Si substrate, indicating that there are more oxygen vacancies in ZnO NRs synthesized on the ITO substrate compared to those synthesized on a Si substrate. Since the oxygen vacancies are higher in ZnO NRs synthesized on an ITO substrate than on a Si substrate, this indirectly confirms that the intensity of the I_G_ value of ZnO NRs synthesized on an ITO substrate is higher than that of ZnO NRs synthesized on a Si substrate. These results are consistent with the measured results presented in Figure 6.

**Figure 7:**
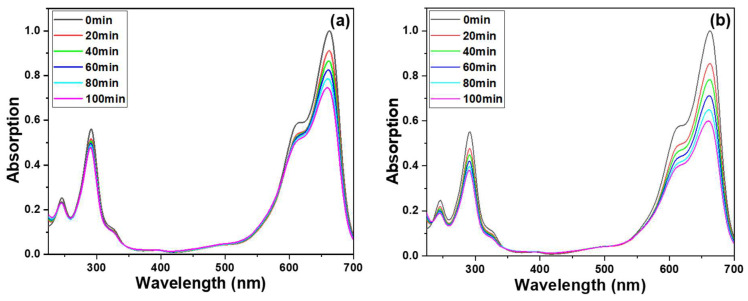

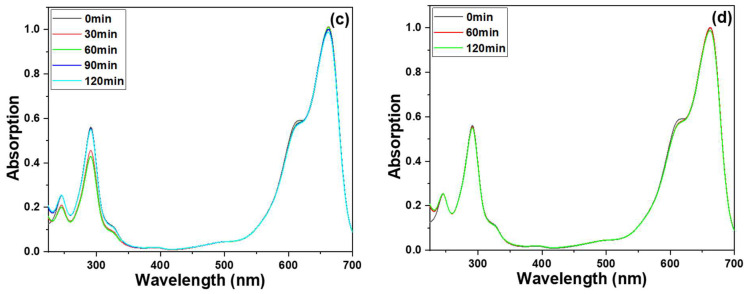
Ultraviolet-visible spectra of treated MB solution under different substrates, (**a**) ZnO NRs synthesized on a Si substrate, (**b**) ZnO NRs synthesized on an ITO substrate, (**c**) only an ITO substrate was used, and (**d**) ZnO NRs synthesized on an ITO substrate and measured at a dark condition. The used MB solution was 5 ppm and 5 mL, and 365 nm ultraviolet light was used as the irradiation light.

To evaluate the photocatalytic activity of ZnO NRs that were prepared, an experiment was conducted to examine the MB degradation using UV-light irradiation. Figure 7a shows the transmittance spectra of MB solutions, where ZnO NRs were grown on Si as the photocatalytic materials. The absorption coefficient spectra of the MB solution treated with ZnO NRs grown on various substrates were also analyzed. The MB molecules exhibited a broad and strong absorption band centered at 665 nm, as well as two sharp bands centered at 246 and 293 nm. To evaluate the degradation results, the intensity of the 665 nm absorption band was used as a reference. As shown in Figure 7a, a typical absorption profile for MB was observed, and the intensities of the entire absorption spectrum and the three absorption bands gradually decreased with increasing treatment time. For example, when ZnO NRs synthesized on a Si substrate were used as photocatalytic materials, the average intensity of the 665 nm band decreased from 91.0% to 86.3%, 82.2%, 78.0%, to 73.7% as the UV light irradiation time increased from 20, 40, 60, 80, to 100 min (Figure 7a). Similarly, when ZnO NRs synthesized on an ITO substrate were used, the intensity of the 665 nm band decreased by 85.4% to 78.3%, 70.9%, 64.7%, and 59.5% as the UV-light irradiation time increased from 20 to 40, 60, 80, and 100 min (Figure 7b). 

These transmittance spectra of MB solutions indicate the degradation effect of ZnO NRs on the MB solution. The experimental results showed that as the different substrates are used, the degradation effects will be different. Even when the concentration of MB is 5 ppm, ZnO NRs synthesized on an ITO substrate can effectively degrade more than 40% of the MB after 100 min of exposure. The findings presented in Figure 7 illustrate that ZnO NRs grown on various substrates exhibit varying crystalline phases and morphologies, resulting in distinct degradation effects on the MB solution. This highlights the importance of substrate selection in the synthesis of ZnO NRs for targeted photocatalytic applications. To establish a causal relationship between the varying crystalline phases and morphologies of ZnO NRs synthesized on different substrates and their distinct effects on MB degradation, an additional experiment was conducted wherein only an ITO substrate was immersed in the prepared MB solution, and the resulting degradation effect is illustrated in Figure 7c. The results depicted in Figure 7c indicate that even after 100 min of the MB degradation experiment using only an ITO substrate, there was only a marginal decrease in the peak intensity at the absorption band of 665 nm. This finding suggests that the degradation rate of pure ITO remains significantly low, indicating a negligible photocatalytic effect of ITO. ITO is a material known for its wide bandgap, ranging from 3.5 to 4.3 eV. Its bandgap determines the energy required for excitation and absorption, with the excitation threshold falling within the ultraviolet region at 3.75 eV, corresponding to a wavelength of 330 nm. Due to the short length of light slope necessary to excite electrons on ITO, achieving a photocatalytic effect on only an ITO substrate can be ignored. This result can prove that the ZnO NRs growing on an ITO substrate has a better MB degradation effect, which is due to the ZnO NRs itself. ZnO NRs grown on an ITO substrate were also tested for decomposition effects in the absence of light. It was observed that MB hardly decomposed when the decomposition time was 60 and 120 min, as Figure 7d shows. This observation further confirms that the decomposition of MB is primarily induced by the photocatalytic effect of UV irradiation on ZnO NRs.

**Figure 8:**
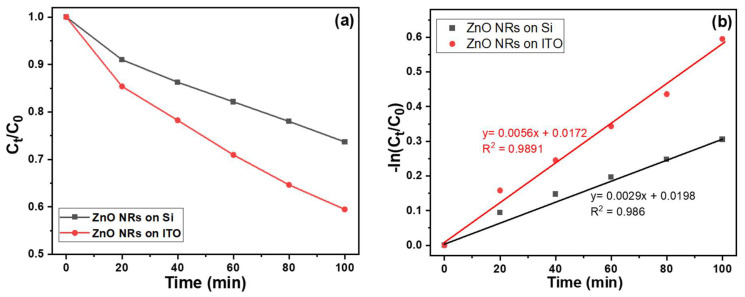
(**a**) Variations of absorption intensities and (**b**) the drawing of Beer–Lambert law for MB solutions under different irradiation times of UV light. The used MB solution was 5 ppm and 5 mL, and 365 nm ultraviolet light was used as the irradiation light.

The measured absorption coefficients can be converted to the absolute concentration of MB in the aqueous solution because the initial concentration of MB is known. Thus, the time evolution of the MB concentration can be calculated in the aqueous solution treated with ultraviolet light. Figure 8 depicts the temporal changes in the concentration of MB, which were derived from the absorption spectra. It is evident from the graph that the decay behavior can be effectively represented by a linear function. The correlation between the concentration of an absorbing substance and its absorbance can be elucidated using the Beer–Lambert law. This principle is extensively employed in the analysis of attenuations in diverse fields such as physical optics involving dilute gases, neutrons, and photons. Moreover, it finds widespread application in chemical analytical measurements. The Beer–Lambert law can be mathematically expressed as follows:

*A* = ln (*I*_0_/*I*) = *abc* (4)

In this equation, *A* represents the absorbance, *I* stands for the intensity of the incident light, and *I*_0_ refers to the intensity of the transmitted light. The variable *a* denotes the absorptivity, which is measured in units of L mol⁻^1^ cm⁻^1^. The parameter *b* represents the path length, which corresponds to the width of the quartz cell containing the detection solution. Additionally, *c* signifies the concentration of the detected material in the solution, expressed in mol L⁻^1^. To acquire normalized values, all the measured absorption intensities at 665 nm are divided by the unmeasured intensity. Figure 8 illustrates the correlation between the duration of irradiation for the MB degradation and the intensity of the absorbance peak at 665 nm. When various substrates were utilized for synthesizing ZnO NRs as the photocatalyst, all the measured data exhibited a linear relationship, as indicated by the fitted lines. Figure 8 demonstrates a clear monotonic decrease in absorbance for the spectral peak at 665 nm with increasing irradiation time. Notably, ZnO NRs synthesized on an ITO substrate demonstrated higher photocatalytic activity in the MB degradation under UV-light exposure compared to ZnO NRs synthesized on a Si substrate, which displayed relatively lower photocatalytic responses. 

The utilization of ZnO NRs synthesized on an ITO substrate as the photocatalyst resulted in a fitted line with a steeper slope when considering the normalized measured intensities. Conversely, when using ZnO NRs synthesized on a Si substrate, the fitted line exhibited a comparatively smaller slope. This observation implies that ZnO NRs synthesized on an ITO substrate exhibit a more pronounced photocatalytic effect in the MB degradation. When comparing the findings with those presented in Figure 2, it is evident that the observed differences in the morphology and specific surface area of ZnO NRs provide further evidence that a larger specific surface area of ZnO NRs is not the sole factor responsible for achieving a more pronounced degradation effect on MB. According to the Beer–Lambert law, the residual ratio (concentration) of MB in the solution is directly proportional to its concentration. However, as shown in Figure 8, there was a linear decrease in the concentration of MB degradation in all ZnO NRs, regardless of the substrates used. Thus, the Equation (2) can be used to express the linearly increasing curves.

*y* = *a* + *b* x (5)

As the substrates used were Si and ITO, the values for *a* value were 0.0029 and 0.0056, and the values for *b* were +0.0198 and +0.00172, respectively. Clearly, the MB degradation exhibited a consistent linear decrease. An essential evaluation parameter in this context is the coefficient of determination, denoted as R^2^. The obtained R^2^ values were 0.986 and 0.9891, respectively. These high R^2^ values indicate that the linear equation aligns well with the experimental data, highlighting a strong correspondence between the model and the observed outcomes.

**Figure 9:**
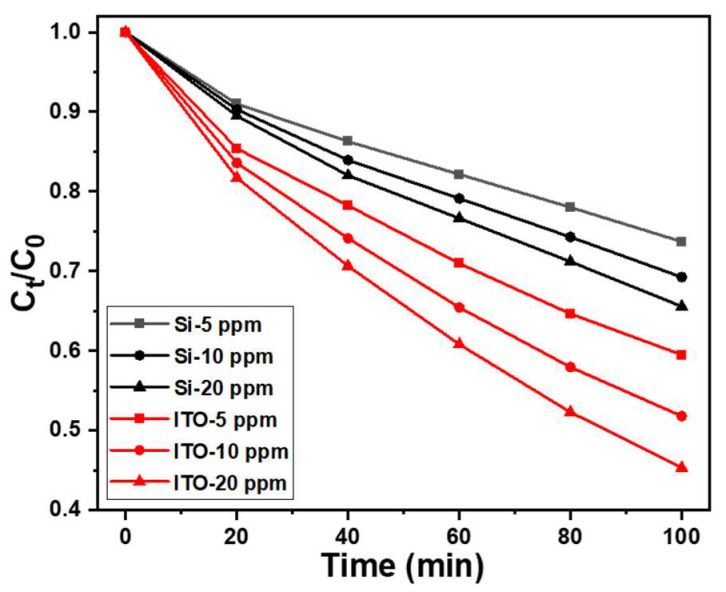
Variations of absorption intensities for MB solutions under different solution concentrations and irradiation times of UV light. The used MB solutions was 5, 10, and 20 ppm and 5 mL, and 365 nm ultraviolet light was used as the irradiation light.

Degradation experiments were conducted using different concentrations of MB, and the results are illustrated in Figure 9. The figure demonstrates that as the concentration increased from 5 ppm to 20 ppm, the degradation rate of ZnO NRs (regardless of the substrate they were grown on) increased significantly. However, it is evident that doubling the MB concentration does not lead to a doubling of the degradation rate. The main reason for this observation is that as the concentration of MB increases, more MB molecules come into contact with the surfaces of ZnO NRs for processing degradation within the same timeframe. While a higher concentration allows for more opportunities for MB molecules to interact with ZnO NRs, the degradation process itself still requires time. Consequently, when there are excessive MB molecules on the surface of ZnO NRs, the degradation efficiency is not solely determined by the solution concentration, but also influenced by the rate of degradation.

Extensive research has been conducted on the adsorption characteristics of MB on various adsorbents. However, when it comes to using photocatalysis for MB degradation, the mechanism is still poorly understood due to the complex chemical structure involved. The process of photocatalytic oxidation treatment of MB involves a photocatalytic oxidation process, where the mechanism remains unclear. During the photocatalytic oxidation treatment of MB, hydroxyl radicals play a crucial role. Initially, these radicals attack the N-S heterocycle group conjugate structure. This occurs because the electron density of sulfhydryl is higher, making it more susceptible to attack. The characteristic peak of a large conjugated system of the N-S heterocycle group in the UV-Vis absorption spectra is observed at 665 nm in the visible region. This wavelength represents a distinct feature of the molecule’s absorption behavior. As a result, MB is degraded into 2-Amino-5-dimethylamino-benzenesulfonic acid anion and Dimethyl-(4-nitro-phenyl)-amine. Under UV irradiation, the excitation of photocatalysis generates hydroxyl radicals, which can further oxidize and mineralize organic pollutants [30]. This process converts them into H_2_O and CO_2_, effectively eliminating the contaminants.

Once the photocatalyst material is exposed to light, the electrons within the raw material undergo a transition from the valence band to the conduction band. This transition generates electron-hole (e^−^/h^+^) pairs on the surface of the photocatalyst. The negatively charged electrons combine with oxygen, resulting in the formation of negative oxygen ions (O^2−^). Simultaneously, the positively charged holes combine with water, leading to the production of hydroxyl radicals (•OH). Both negative oxygen ions and hydroxyl radicals are highly reactive substances with low chemical stability. When organic substances, specifically hydrocarbons, come into contact with the surface of the photocatalyst, they react separately with negative oxygen ions and hydroxyl radicals. Subsequently, these reactions cause the hydrocarbons to recombine and form carbon dioxide (CO_2_) and water (H_2_O). Following the completion of the reaction, the electrons and holes return to their original positions within the photocatalyst, awaiting the next combination with oxygen and water.

To enhance the photocatalytic properties for MB degradation, electrons are essential for the nanomaterials to exhibit their effectiveness. ZnO NRs grown on ITO exhibit higher defect levels in the visible light range, as indicated by their PL spectra. Moreover, the lower energy band gap observed in these NRs further confirms the presence of increased defects. These higher defect levels can lead to an augmented number of electrons, thereby enhancing the degradation efficiency. Thus, there are three reasons for the improved effect of using ITO as substrate. Firstly, ZnO NRs grown on an ITO substrate exhibit a higher density of defects. Consequently, when light irradiates this substrate, electrons and holes are more readily decomposed and acquired, resulting in a pronounced degradation of MB. However, the second reason is that when ZnO NRs are synthesized on an ITO substrate, as mentioned earlier, many electrons in an ITO substrate can provide for MB degradation and these electrons can be conducted through ZnO NRs. Thirdly, ZnO NRs exhibit better crystallinity and fewer defects when they are synthesized on a Si substrate. On the other hand, ZnO NRs synthesized on an ITO substrate tend to have more defects. As a result, an ITO substrate possesses a higher concentration of electrons compared to ZnO NRs. Figure 7c demonstrates that electrons on an ITO substrate lack photocatalytic characteristics. During the MB-degradation process, electrons are essential, and the ITO substrate has a greater number of electrons to enhance the efficiency of MB degradation. Therefore, ZnO NRs synthesized on an ITO substrate leads to a better MB degradation efficiency due to the increased availability of electrons. 

## 4. Conclusions

In our study, that ZnO NRs synthesized on a Si substrate only exhibited the (002), (101), and (102) planes were observed. However, the presence of (002), (101), and (102) planes alongside (100) indicates the formation of ZnO NRs on ITO substrate with a more complex crystal structure, which may be caused by the formation of more defects in ZnO NRs synthesized on an ITO substrate. The SEM top images consistently revealed hexagonal nanorods along the lengths of ZnO NRs synthesized on both substrates, indicating a hexagonal crystal structure and a preference for growth along the *c*-axis direction. The decrease in the energy band gap serves as indirect evidence suggesting the presence of a greater number of defects, resulting in a lower growth rate. From the SEM images, ZnO NRs synthesized on an ITO substrate had slower growth rate of ZnO NRs than that on an ITO substrate. The reason can be attributed to a high concentration of carrier electrons on the surface, which affects the concentration of OH^−^ ions. This phenomenon is recognized as the primary reason behind the observed discrepancy in growth rates between the two substrates. The intensity of this absorption band at 665 nm was utilized as a reference to determine the efficiency of MB degradation. In the PL spectrum of ZnO NRs synthesized on an ITO substrate, a distinct green emission was observed. This green emission can be attributed to various defects present in the ZnO nanomaterials. XPS analyses revealed that the OII peak areas were 24.1% and 39.1% for ZnO NRs synthesized on Si and ITO substrates, respectively. This finding further confirms that ZnO NRs synthesized on an ITO substrate exhibited a higher density of defects compared to those synthesized on a Si substrate. Interestingly, the analyses results also reveal that ZnO NRs synthesized on an ITO substrate demonstrated a more pronounced degradation effect on MB compared to those synthesized on a Si substrate. Additionally, ZnO NRs synthesized on an ITO substrate were observed to display a higher MB degradation efficiency compared to those synthesized on a Si substrate. The higher defect levels in the PL spectrum and the lower energy band gap can cause three reasons to contribute to this phenomenon higher degradation efficiency. Firstly, ZnO NRs grown on an ITO substrate exhibited a higher density of defects, which can act as active sites to enhance the overall degradation efficiency. Secondly, an ITO substrate provided a higher concentration of electrons to react with MB molecules, facilitating their degradation. Lastly, ZnO NRs synthesized on an ITO substrate tend to possess more defects and a higher concentration of electrons, further augmenting their capability for MB degradation. This combination of defects and electron concentration contributes to the superior MB degradation efficiency observed in ZnO NRs synthesized on an ITO substrate.

## Data Availability

Not applicable.
